# Association of the Infant Gut Microbiome With Early Childhood Neurodevelopmental Outcomes: An Ancillary Study to the VDAART Randomized Clinical Trial

**DOI:** 10.1001/jamanetworkopen.2019.0905

**Published:** 2019-03-22

**Authors:** Joanne E. Sordillo, Susan Korrick, Nancy Laranjo, Vincent Carey, George M. Weinstock, Diane R. Gold, George O’Connor, Megan Sandel, Leonard B. Bacharier, Avraham Beigelman, Robert Zeiger, Augusto A. Litonjua, Scott T. Weiss

**Affiliations:** 1 Department of Population Medicine, Harvard Medical School, Harvard Pilgrim Health Care Institute, Boston, Massachusetts; 2 Department of Environmental Health, Harvard T.H. Chan School of Public Health, Boston, Massachusetts; 3 Channing Division of Network Medicine, Harvard Medical School, Brigham and Women’s Hospital, Boston, Massachusetts; 4 The Jackson Laboratory for Genomic Medicine, Farmington, Connecticut; 5 Department of Medicine, Boston University School of Medicine, Boston, Massachusetts; 6 Division of Allergy, Immunology, and Pulmonary Medicine, Washington University School of Medicine in St Louis, St Louis, Missouri; 7 Department of Allergy and Research and Evaluation, Kaiser Permanente Southern California Region, San Diego and Pasadena; 8 Department of Pediatrics, University of Rochester, Rochester, New York

## Abstract

**Importance:**

In animal models, the early life gut microbiome influences later neurodevelopment. Corresponding data in human populations are lacking.

**Objective:**

To study associations between the gut microbiome in infants and development at preschool age measured by the Ages and Stages Questionnaire, third edition (ASQ-3).

**Design, Setting, and Participants:**

This ancillary cohort study of the Vitamin D Antenatal Asthma Reduction Trial (VDAART) used data from 715 participants who had development assessed at 3 years of age by the ASQ-3, which included scores in 5 domains (gross motor skills, fine motor skills, problem solving, communication, and personal and social skills). A total of 309 stool samples were collected from infants aged 3 to 6 months for microbiome analysis using *16S rRNA* gene sequencing.

**Exposures:**

Infant gut microbiome.

**Main Outcomes and Measures:**

Continuous ASQ-3 scores and typical vs potential delay in the 5 developmental domains. Factor scores for bacterial coabundance groups were used as predictors in regression models of continuous ASQ-3 scores. Logistic regression was used to examine bacterial coabundance scores and odds of scoring below the threshold for typical development. Multivariate analysis examined the abundance of individual taxa and ASQ-3 scores.

**Results:**

The 309 participants (170 [55.0%] male) with ASQ-3 scores and stool samples were ethnically diverse (136 [44.0%] black, 41 [13.3%] Hispanic, 86 [27.8%] white, and 46 [14.9%] other race/ethnicity); the mean (SD) age at ASQ-3 assessment was 3.0 (0.07) years. Coabundance scores dominated by Clostridiales (Lachnospiraceae genera and other, unclassified Clostridiales taxa) were associated with poorer ASQ-3 communication (β, –1.12; 95% CI, −2.23 to −0.01; *P* = .05) and personal and social (β, –1.44; 95% CI, −2.47 to −0.40; *P* = .01) scores and with increased odds of potential delay for communication (odds ratio [OR], 1.69; 95% CI, 1.06 to 2.68) and personal and social skills (OR, 1.96; 95% CI, 1.22 to 3.15) per unit increase in coabundance score. The *Bacteroides*-dominated coabundance grouping was associated with poorer fine motor scores (β, –2.42; 95% CI, −4.29 to −0.55; *P* = .01) and with increased odds of potential delay for fine motor skills (OR, 1.52; 95% CI, 1.07 to 2.16) per unit increase in coabundance score. Multivariate analysis detected similar family-level and order-level associations.

**Conclusions and Relevance:**

These findings suggest an association between infant gut microbiome composition and communication, personal and social, and fine motor skills at age 3 years. The majority of associations were driven by taxa within the order Clostridiales.

## Key Points

**Question:**

Is the gut microbiome in infancy associated with neurodevelopment in children at preschool age?

**Findings:**

In this ancillary cohort study of the Vitamin D Antenatal Asthma Reduction Trial (VDAART) of 309 infants with fecal flora samples and development at preschool age measured by the Ages and Stages Questionnaire, a statistically significant association was observed between the gut microbiome in infancy and fine motor, communication, and personal and social skills at 3 years of age.

**Meaning:**

These epidemiologic findings appear to support the hypothesis that early life gut microbiota are associated with neurodevelopmental outcomes in childhood.

## Teaser

This ancillary cohort study of the Vitamin D Antenatal Asthma Reduction Trial (VDAART) examines the association of the gut microbiome in infancy with development of motor skills, problem solving, communication, and personal and social skills at preschool age.

## Introduction

Emerging evidence suggests that infant gut microbes contribute to neurodevelopment. In humans, colonization of the infant gut likely begins before birth,^1,2^ with a rapid increase in microbial burden during the first few weeks of life.^3^ Microbial communities in the infant gut remain remarkably dynamic until about 3 years of age, when more stable microbial profiles begin to emerge.^4^ Microbes colonizing the infant gut are important regulators of a number of physiological processes that are critical to neurodevelopment, such as nutrient uptake and immune system development.^5^ The microbiome’s pivotal role in immune system development and regulation is a hypothesized mechanism for its association with cognitive or behavioral disorders, many of which correlate with immune dysfunction including autism spectrum disorders (ASDs), a phenotype characterized by deficits in communication and social interaction.^6,7^ Experimental animal models support a potential link between the gut microbiome and autism. Germ-free mice show phenotypic traits consistent with autism, including deficits in social interaction that can be reversed by colonization after weaning.^8^

Similarly, in human populations, differences in gut microbial composition have been associated with ASDs. In a comparison of 20 children and adolescents with ASD and 20 neurotypical control individuals between the ages of 3 and 16 years, those with ASD had lower abundances of the gut microbiota *Coprococcus, Prevotella*, and unclassified Veillonellaceae.^9^ A more recent study also found differences in gut microbial composition among children between the ages of 3 and 12 years with ASD and gastrointestinal symptoms compared with 41 typically developing controls also with gastrointestinal symptoms.^10^ However, in cross-sectional analyses, it is difficult to know the directionality of observed associations. Furthermore, these studies were performed in older children, in whom the gut microbiome is expected to be established. In one of the few published prospective human studies assessing the microbiome and neurodevelopment, greater relative abundance of *Bacteroides* at 1 year was associated with better expressive and receptive language in 69 neurotypical 2-year-old children.^11^

To prospectively assess the hypothesized association of early life gut microbiota with subsequent child neurodevelopment, we leveraged data available from the Vitamin D Antenatal Asthma Reduction Trial (VDAART),^12^ which assessed the effect of vitamin D supplementation in pregnancy on development of asthma-related phenotypes in offspring. Specifically, we assessed associations between the gut microbiome in infants and developmental outcomes at preschool age measured by the Ages and Stages Questionnaire, third edition (ASQ-3).^15^ The ASQ-3 is used to characterize a young child’s development across a range of skills. Of note, poor performance results on the ASQ-3 (particularly on communication skills) for children from ages 16 to 30 months has been shown to be sensitive (but not specific) for diagnosis of ASD.^13^ We examined the association between community composition of the gut microbiome at age 3 to 6 months and parent-reported ASQ-3 scores of children at age 36 months.

## Methods

### Clinical Trial and Ancillary Study

This study was an ancillary study of VDAART, a 2-arm, double-blind, placebo-controlled, randomized clinical trial (n = 810 infants) to determine whether higher vitamin D intake and levels in pregnant mothers prevents childhood asthma and allergy in their offspring (eMethods in the Supplement).^12^ The VDAART protocol and the VDAART flora ancillary study were approved by the institutional review boards at each participating institution and at the Brigham and Women’s Hospital. All women provided written informed consent, and the data were deidentified. This study followed the Strengthening the Reporting of Observational Studies in Epidemiology (STROBE) reporting guideline for cohort studies.^14^

### Ages and Stages Questionnaire

At approximately 3 years of age, study researchers administered the ASQ-3^15^ to 715 primary caregivers during a study clinic visit or phone interview. The ASQ-3 assesses 5 developmental domains: gross motor skills, fine motor skills, problem-solving ability, communication, and personal and social skills. Each domain is assessed by 6 questions ascertaining achievement of relevant skills and answered as yes (10 points), sometimes (5 points), or not yet (0 points). Scores for individual items are summed to give an overall continuous score for each of the 5 domains (possible range, 0-60). In addition to using ASQ-3 scores as continuous outcomes, we categorized the ASQ-3 scores according to a priori recommendations^15^ of 3 categories (typical development, need for monitoring, or need for further assessment) (eTable 1 in the Supplement). We collapsed categories into a binary variable (those with typical development vs those with a need for monitoring or further assessment [ie, potential atypical or delayed development]).

### Stool Sample Collection and Sequencing of Bacterial *16S rRNA* DNA

Stool samples (n = 333) were collected in infancy at ages 3 to 6 months, and 309 of 333 participants (92.8%) with infant stool collection also had ASQ-3 outcomes at age 3 years. DNA extractions were performed on stool samples, and the bacterial *16S rRNA* gene (V3 to V5 hypervariable regions) was amplified. The eMethods in the Supplement gives additional information about stool sample collection, sequencing, quality filtering, and bioinformatic processing of sequences.

### Covariates

On the basis of a priori considerations, we adjusted all models for age at ASQ-3 assessment, cholecalciferol (vitamin D) treatment group, clinical site, and other potential confounders described below. Mode of delivery, child’s sex, and antibiotic administration in the first days of life were abstracted from medical records. Gestational age at birth was calculated based on estimated conception date (using ultrasonography) and date of delivery. Maternal age, marital status, educational level, family income, and infant race/ethnicity were assessed by enrollment questionnaires (eMethods in the Supplement). Breastfeeding was assessed by maternal questionnaire 6 months after birth.

### Statistical Analysis

Primary analyses focused on associations of bacterial coabundance groupings with continuous ASQ-3 scores. To explore potential clinical implications of these primary findings, analyses were repeated with dichotomized scores (above or below the ASQ-3 threshold for typical development). Additional secondary analyses considered 2 other facets of the microbiome: microbiome diversity and abundances of each specific taxon. For linear and logistic regression analyses, 2-tailed *P* ≤ .05 was considered to be the threshold for statistical significance. Multivariate (MaAsLin) analysis for individual taxa provides both a nominal *P* value and a false discovery rate–adjusted *P* value (*q* value). To minimize false discovery rates, we used dimensionality reduction (bacterial coabundance groups) to characterize the microbiome for our primary analysis. We chose not to use classic multiple comparison correction techniques (ie, Bonferroni) because these would be too conservative, given the correlated nature of the outcomes^16^ and the expected overlap of findings across different analytical approaches.

#### Coabundance Groupings

To capture coabundance groupings (or factor representations) of bacterial taxa within the infant gut, we computed Spearman rank correlation coefficients for the top 25 taxa identified using the CCREPE package in R biostatistical software (R Foundation) and used the correlation matrix for principal factor analysis (with varimax rotation). Coabundance groupings have been published previously^17^ (eTable 2 in the Supplement). In brief, factor loadings were applied to *16S rRNA* sequencing counts to compute an individual’s factor score for each bacterial coabundance group. Scores for bacterial coabundance groupings were used as independent variables in linear regression models of continuous ASQ-3 scores. Because vitamin D has been correlated with infant gut microbial status in other studies,^18^ including VDAART,^17^ we did sensitivity analyses to assess the potential for vitamin D treatment group to modify microbiome associations with ASQ-3 scores. Specifically, we added an interaction term between vitamin D treatment group and bacterial coabundance factor scores to models of ASQ-3 outcomes. Because of potential variability in microbiome by study site, we also assessed the potential for location to modify associations. Adjusted logistic regression models were used to determine the odds of scoring below the typical development threshold.

#### Diversity of the Microbiome

We computed diversity of the infant gut microbiome using the Shannon diversity index (representing both the evenness of taxonomic distribution and the total number of taxa) and used it as a predictor for continuous ASQ-3 scores in adjusted linear regression models.

#### Multivariate Associations

Because the presence or absence of less abundant taxa may be associated with health outcomes, we also did analyses using multivariate associations with linear models (MaAsLin)^19^ to capture additional taxa not identified using coabundance groupings (eTables 3-5 in the Supplement). As a predictor of microbial taxa, we dichotomized ASQ scores at the threshold for typical development.

## Results

### Participants

The 309 participants with ASQ-3 scores and gut microbiome sequencing had similar characteristics compared with the 406 VDAART participants who had ASQ-3 measures but no gut microbiome assessment (Table 1). Children in this analysis were primarily full term at birth (mean [SD] gestational age, 39.1 [1.9] weeks), and 170 (55%) were male. Consistent with VDAART’s focus on inner city minority communities, participants were racially/ethnically diverse: 136 (44%) black, 41 (13.3%) Hispanic, 86 (27.8%) white, and 46 (14.9%) other race/ethnicity. Most children were delivered vaginally and were breastfed in the first 6 months of life, which are both factors that may be associated with the infant microbiome (Table 1).

**Table 1.** Characteristics of Mothers and Infants in the VDAART Cohort With ASQ-3 Assessments^a^

Characteristic	Infants With ASQ-3 Assessment at 3 y of Age	
	Without Infant Fecal Flora Sample (n = 406)	With Fecal Flora Sample (n = 309)
Gestational age, mean (SD), w	38.9 (2.0)	39.1 (1.9)
Child’s race/ethnicity		
Black	180 (44.3)	136 (44.0)
Hispanic	54 (13.3)	41 (13.3)
White	104 (25.6)	86 (27.8)
Other	68 (16.8)	46 (14.9)
Male	210 (51.7)	170 (55.0)
Breastfed first 6 mo^b^	207 (56.3)	169 (55.0)
Cesarean delivery	115 (28.3)	100 (32.4)
Infant antibiotic therapy, first days of life^b^	42 (10.4)	24 (7.8)
Income <$30 000/y	117 (28.8)	94 (30.4)
Maternal educational level		
Did not graduate high school	56 (13.8)	36 (11.7)
Graduated high school or technical school	114 (28.1)	92 (29.8)
Some college or junior college	93 (22.9)	72 (23.3)
Graduated college	89 (21.9)	56 (18.1)
Graduate school	54 (13.3)	53 (17.2)
Maternal marital status of married	183 (45.1)	142 (46.0)
ASQ-3 score, mean (SD)^c^		
Gross motor skills	56.0 (6.4)	56.1 (7.3)
Fine motor skills	43.8 (14.9)	42.5 (15.7)
Communication	53.3 (7.9)	52.7 (8.9)
Personal and social skills	54.1 (7.9)	53.9 (8.2)
Problem solving skills	52.9 (9.8)	52.7 (10.0)

Abbreviations: ASQ-3, Ages and Stages Questionnaire, third edition; VDAART, Vitamin D Antenatal Asthma Reduction Trial.

^a^ Data are given as number (percentage) of individuals, unless otherwise indicated. *P* > .05 for all comparisons.

^b^ Percentage based on nonmissing values. Breastfeeding data missing for 38 individuals in the group without fecal flora samples and for 2 in the group with fecal flora samples; data on infant antibiotic therapy missing for 1 individual in the group without fecal flora samples.

^c^ Possible score ranges from 0 to 60; higher score indicates better performance.

### ASQ-3 Scores

Mean (SD) age at ASQ assessment was 3.0 (0.07) years. The medians (ranges) for the ASQ-3 scores were as follows: gross motor (60 [10-60]), fine motor (45 [0-60]), communication (55 [0-60]), personal and social skills (55 [20-60]), and problem solving (60 [5-60]). Most 3-year-old children in the study met the threshold for typical neurodevelopment. However, there were children whose scores suggested the need for monitoring or further evaluation: 31 of 309 (10%) for gross motor skills, 70 (23%) for fine motor skills, 46 (15%) for problem solving, 31 (10%) for communication, and 32 (10%) for personal and social skills. Of those scoring below the typical development threshold in at least 1 domain (117 of 309 participants [38%]); of the 117 participants, 67 (57%) were below the threshold in 1 domain, 24 (21%) in 2 domains, 15 (13%) in 3 domains, 7 (6%) in 4 domains, and 4 (3%) in all 5 domains.

### Demographic Characteristics and ASQ-3 Scores

The ASQ-3 scores were associated with a number of demographic and early life characteristics (Table 2). Older gestational age was associated with higher ASQ-3 scores in 4 developmental domains. Females had higher personal and social scores and higher fine motor scores but lower gross motor scores than males. Higher maternal education was associated with better fine motor, problem-solving, and personal and social skills. Cesarean delivery was associated with decreased ASQ scores, and breastfeeding did not show any statistically significant associations. Vitamin D treatment during pregnancy was not associated with ASQ-3 scores. Except for gross motor skills, ASQ-3 scores were higher at the St Louis, Missouri, and San Diego, California, sites compared with the Boston, Massachusetts, clinical site.

**Table 2.** Association of Maternal and Infant Characteristics and Infant Gut Microbiome With ASQ-3 Scores at 3 Years of Age

Infant Characteristic (n = 307)	Multivariable Regression Models for ASQ-3 Assessments at 3 Years of Age										
	Gross Motor Skills		Fine Motor Skills		Communication		Personal and Social Skills		Problem Solving Skills		
	β (95% CI)	*P* Value	β (95% CI)	*P* Value	β (95% CI)	*P* Value	β (95% CI)	*P* Value	β (95% CI)	*P* Value
Gestational age, wk	0.89 (0.40 to 1.38)	.004	1.06 (0.06 to 2.06)	.04	0.71 (0.15 to 1.28)	.01	0.22 (−0.31 to 0.76)	.41	0.68 (0.01 to 1.35)	.05
Child’s race/ethnicity^a^										
Hispanic	−1.52 (−4.58 to 1.53)	.33	4.22 (−2.03 to 10.47)	.19	0.07 (−3.47 to 3.61)	.97	−2.60 (−5.91 to 0.71)	.12	−0.57 (−4.74 to 3.61)	.79
White	−1.35 (−4.13 to 1.43)	.34	4.15 (−1.54 to 9.85)	.15	−1.72 (−4.95 to 1.50)	.29	−2.43 (−5.45 to 0.59)	.11	−1.66 (−5.46 to 2.14)	.39
Other	−0.97 (−4.11 to 2.17)	.55	6.97 (0.55 to 13.40)	.03	−2.07 (−5.71 to 1.57)	.26	−3.03 (−6.43 to 0.38)	.08	−0.09 (−4.38 to 4.20)	.97
Female (vs male)	−1.59 (−3.30 to 0.12)	.07	4.25 (0.76 to 7.74)	.02	1.38 (−0.60 to 3.36)	.17	1.66 (−0.19 to 3.52)	.08	0.89 (−1.45 to 3.22)	.46
Breastfed in first 6 mo	0.55 (−1.46 to 2.56)	.59	1.82 (−2.29 to 5.93)	.38	1.72 (−0.61 to 4.05)	.15	0.50 (−1.68 to 2.68)	.65	0.66 (−2.09 to 3.40)	.64
Cesarean delivery	−0.87 (−2.69 to 0.94)	.34	−3.66 (−7.38 to 0.05)	.05	−2.37 (−4.47 to −0.27)	.03	−2.23 (−4.19 to −0.26)	.03	−1.18 (−3.66 to 1.29)	.35
Infant antibiotic therapy in first days of life	3.07 (−0.46 to 6.61)	.09	−1.17 (−8.41 to 6.07)	.75	0.60 (−3.50 to 4.70)	.77	0.12 (−3.72 to 3.96)	.95	−0.15 (−4.99 to 4.68)	.95
Age at ASQ-3 assessment, y	−8.15 (−20.15 to 3.86)	.18	2.49 (−22.09 to 27.07)	.84	−0.46 (−14.38 to 13.46)	.95	−3.92 (−16.95 to 9.11)	.55	−13.10 (−29.51 to 3.31)	.12
Maternal educational level	0.06 (−0.87 to 0.99)	.90	2.42 (0.52 to 4.32)	.01	0.97 (−0.11 to 2.05)	.08	1.24 (0.23 to 2.25)	.02	1.59 (0.33 to 2.86)	.01
Maternal marital status (married vs not married)	0.80 (−1.49 to 3.08)	.49	−1.39 (−6.07 to 3.28)	.56	−1.00 (−3.64 to 1.65)	.46	−0.56 (−3.04 to 1.92)	.66	−0.44 (−3.56 to 2.68)	.78
Income <$30 000/y	−1.44 (−3.48 to 0.60)	.17	1.57 (−2.61 to 5.75)	.46	−1.22 (−3.58 to 1.15)	.31	−1.40 (−3.61 to 0.82)	.22	−2.74 (−5.53 to 0.05)	.05
Maternal age	−0.07 (−0.26 to 0.12)	.49	−0.11 (−0.49 to 0.28)	.59	0.009 (−0.21 to 0.23)	.94	−0.08 (−0.28 to 0.13)	.46	−0.05 (−0.31 to 0.21)	.70
Vitamin D treatment arm, 4000 IU	−0.57 (−2.23 to 1.09)	.50	−2.61 (−6.01 to 0.80)	.13	0.38 (−1.55 to 2.31)	.70	−0.05 (−1.86 to 1.75)	.95	−0.60 (−2.87 to 1.67)	.60
Clinical site^b^										
St Louis, Missouri	−0.25 (−2.50 to 2.00)	.83	7.56 (2.94 to 12.17)	.001	5.80 (3.19 to 8.41)	<.001	4.20 (1.76 to 6.65)	<.001	3.06 (−0.02 to 6.14)	.05
San Diego, California	0.79 (−1.83 to 3.42)	.55	2.39 (−2.99 to 7.76)	.38	5.58 (2.54 to 8.62)	<.001	4.59 (1.74 to 7.44)	.002	3.18 (−0.41 to 6.77)	.08
Bacterial coabundance scores^c^										
Factor 1	−0.55 (−1.50 to 0.41)	.26	−1.16 (−3.12 to 0.80)	.24	−1.12 (−2.23 to −0.01)	.05	−1.44 (−2.47 to −0.40)	.01	0.42 (−0.89 to 1.72)	.53
Factor 2	0.68 (−0.44 to 1.79)	.23	0.63 (−1.64 to 2.92)	.58	0.65 (−0.64 to 1.94)	.32	0.13 (−1.08 to 1.34)	.83	0.45 (−1.07 to 1.97)	.56
Factor 3	−0.21 (−1.12 to 0.71)	.66	−2.42 (−4.29 to −0.55)	.01	−0.03 (−1.09 to 1.03)	.96	−0.07 (−1.06 to 0.92)	.89	0.53 (−0.72 to 1.77)	.41
Factor 4	0.21 (−0.86 to 1.28)	.70	1.33 (−0.87 to 3.52)	.23	0.81 (−0.44 to 2.05)	.20	0.66 (−0.50 to 1.82)	.26	0.03 (−1.43 to 1.50)	.96

Abbreviation: ASQ-3, Ages and Stages Questionnaire, third edition.

^a^ Reference group: black race/ethnicity.

^b^ Compared with Boston, Massachusetts, clinical site.

^c^ Factor 1: positive for Lachnospiraceae and unclassified Clostridiales and negative for *Bacteroides*; factor 2: positive for *Klebsiella* and *Enterobacter*; factor 3: positive for *Bacteroides* and negative for *Escherichia* and *Shigella* and *Bifidobacterium*; and factor 4: positive for *Veillonella* and *Clostridium*.

### Infant Gut Microbiome

Factor analysis of the 25 most abundant bacterial taxa in the infant gut (eTable 2 in the Supplement) revealed a 4-factor solution based on a scree plot. The first factor captured coabundance of Lachnospiraceae genera (positive loadings, 0.4-0.8) and unclassified Clostridiales taxa (loading, 0.65), both of which are in the Firmicutes phylum and Clostridiales order. *Bacteroides* was negatively associated with this Lachnospiraceae and unclassified Clostridiales taxa coabundance grouping (loading, −0.31). For the second factor, positive loadings for *Klebsiella* (loading, 0.46), *Enterobacter* (loading, 0.86), and unclassified Enterobacteriaceae (loading, 0.76) were most substantial. The third factor was defined mainly by *Bacteroides* abundance (loading, 0.70), with negative factor loadings for *Escherichia*/*Shigella* (loading, –0.44) and *Bifidobacterium* (loading, –0.47). The fourth factor showed substantial positive loadings for *Veillonella* (loading, 0.66) and *Clostridium* (loading, 0.36)*.*

### Gut Microbiome and Continuous ASQ-3 Scores

Bacterial coabundance scores were associated with continuous ASQ-3 scores in 3 domains: fine motor skills, communication skills, and personal and social skills (Table 2). Scores for factor 1 (representing an increased abundance of Lachnospiraceae and unclassified Clostridiales taxa and decreased abundance of *Bacteroides*) were associated with lower communication (β, –1.12; 95% CI,−2.23 to −0.01; *P* = .05), and personal and social (β, –1.44; 95% CI, −2.47 to −0.40; *P* = .01) scores. For both communication skills and personal and social skills, a 1-unit increase in factor 1 was associated with approximately a 1-unit decrease in ASQ-3 score. Factor 3 (*Bacteroides* dominated) was associated with a 2.42-unit decrease (95% CI, −4.29 to −0.55; *P* = .01) in fine motor scores. Our multivariable regression models did not detect any associations between bacterial coabundance scores (factors) and development of gross motor or problem-solving skills.

In sensitivity analyses, we identified the potential for vitamin D treatment to modify associations between microbiome and ASQ-3 scores. In participants who received 4000 IU of prenatal vitamin D treatment, factor 4 (*Veillonella* dominated) was associated with improved communication scores (β, 2.21; 95% CI, 0.58 to 3.84), whereas no association between factor 4 and communication scores was observed for those in the 400-IU vitamin D group (β, –0.91; 95% CI, –2.70 to 0.90) (interaction *P* = .01). Significant adverse overall associations of bacterial coabundance factor 1 with personal and social skills scores and factor 3 with fine motor skills scores were attenuated (*P* = .09 to *P* = .10 for interaction terms) by 4000-IU vitamin D treatment. For personal and social skills, factor 1 was associated with a larger reduction in scores in the 400-IU vitamin D treatment group (β, –2.41; 95% CI, –3.94 to –0.89) compared with the 4000-IU vitamin D treatment group (β, –0.71; 95% CI, –2.03 to 0.61) (interaction *P* = .09). For fine motor skills, factor 3 showed a greater adverse association in the 400-IU vitamin D treatment group (β, –3.91; 95% CI, –6.48 to –1.34) compared with the 4000-IU vitamin D treatment group (β, –0.85; 95% CI, –3.48 to 1.77) (interaction *P* = .10). There was no evidence that study site modified associations.

### Bacterial Coabundance and Dichotomous ASQ-3 Scores

In addition to examining ASQ-3 scores as continuous outcomes, we assessed the odds of scoring below the typical development threshold per unit increase in bacterial coabundance scores for the 3 domains (fine motor skills, communication, and personal and social skills) that were associated with microbiome composition in the continuous analyses (Table 2 and Table 3). Factor 1 was associated with increased odds of scoring below the typical development threshold for both communication (odds ratio, 1.69; 95% CI, 1.06-2.68) and personal and social skills (odds ratio, 1.96; 95% CI, 1.22-3.15). Factor 3 was associated with increased odds of below-threshold fine motor skills scores (odds ratio, 1.52; 95% CI, 1.07-2.16).

**Table 3.** Microbiome Bacterial Coabundance Factors in the Infant Gut and Odds of ASQ-3 Score Below the Typical Development Threshold

Bacterial Coabundance Score at 3-6 mo of Age^a^	ASQ-3 Developmental Score at 3 Years of Age, OR (95% CI)^b^		
	Fine Motor Skills	Communication Skills	Personal and Social Skills
Factor 1	1.28 (0.92-1.78)	1.69 (1.06-2.68)	1.96 (1.22-3.15)
Factor 2	1.13 (0.76-1.68)	0.97 (0.54-1.75)	0.93 (0.48-1.79)
Factor 3	1.52 (1.07-2.16)	1.05 (0.64-1.73)	1.26 (0.72-2.21)
Factor 4	0.80 (0.54-1.19)	0.88 (0.50-1.55)	1.02 (0.60-1.73)

Abbreviations: ASQ-3, Ages and Stages Questionnaire, third edition; OR, odds ratio.

^a^ The numbers of infants with a below typical development threshold among all infants in each group were 70 of 307 for fine motor skills, 32 of 307 for communication skills, and 31 of 307 for personal and social skills. Factor 1: positive for Lachnospiraceae and unclassified Clostridiales and negative for *Bacteroides*; factor 2: positive for *Klebsiella* and *Enterobacter*; factor 3: positive for *Bacteroides* and negative for *Escherichia* and *Shigella* and *Bifidobacterium*; and factor 4: positive for *Veillonella and Clostridium*.

^b^ All models were adjusted for gestational age, race/ethnicity, sex, breastfeeding in first 6 months of life, cesarean delivery, age at ASQ-3 assessment, maternal educational level, maternal marital status, maternal age, low annual income (<$30 000), antibiotic therapy in the first days of life, treatment group, and clinical site; ORs are for a 1-unit increase in factor score.

### Diversity of the Gut Microbiome and ASQ-3 Scores

The mean (SD) Shannon diversity index of the infant gut microbiome was 2.2 (0.46). Overall, the Shannon diversity index was not associated with ASQ-3 scores (gross motor skills: β, −0.59 [95% CI, −2.65 to 1.47]; fine motor skills: β, −0.44 [95% CI, −4.70 to 3.83]; communication: β, −1.09 [95% CI, −3.49 to 1.31]; and problem-solving skills: β, −0.97 [95% CI, −3.77 to 1.84]). We observed an association of increased Shannon diversity index with lower personal and social skills, but it was not statistically significant (β, −1.99; 95% CI, −4.23 to 0.26; *P* = .11).

### Multivariate Analyses of Gut Microbiome and ASQ-3 Scores

Results of multivariate associations analysis with linear models (MaAsLin) for the ASQ-3 scores of communication skills and personal and social skills were consistent with the bacterial coabundance group analysis in that the same bacterial taxonomic orders and families were ranked at the top of the list for associations with ASQ-3 scores (eTable 3 and eTable 4 in the Supplement), although the specific (genus-level) taxa were often different. Children with typically developing communication skills had lower abundance of Clostridiales, in particular Lachnospiraceae *Ruminococcus* (MaAsLin coefficient, −0.065; *P* = .04) (eTable 3 in the Supplement). For the personal and social skills ASQ-3 domain, 3 of the 6 top MaAsLin results suggest that taxa within the Clostridiales order have lower abundance in typically developing children (eTable 4 in the Supplement). Although both the bacterial coabundance group analysis and MaAsLin suggest that children with typically developing communication (with higher ASQ-3 scores) have lower abundance of Clostridiales, the specific genera ranking at the top of the MaAsLin results were different from those in the bacterial coabundance analysis. For typical fine motor skills development, results of MaAsLin did not include the specific genera represented by the factor-3 bacterial coabundance group but showed associations with lower Enterobacteriaceae (notably *Klebsiella*: MaAsLin coefficient, −0.033; *P* < .001) (eTable 5 in the Supplement).

## Discussion

We explored associations between the infant gut microbiome and early childhood neurodevelopment measured by parental report on the ASQ-3. Whereas animal model studies show a direct connection between early life gut microbiota and neurobehavioral development,^20^ data in human populations are lacking. Our findings suggest that the infant gut microbiome may be associated with subsequent development of communication, personal and social, and fine motor skills in typical developing 3-year-old children and with odds of possible developmental delays. Most of the observed associations were attributable to taxa within the order Clostridiales. The Clostridiales taxa–associated coabundance factor included negative loadings for *Bacteroides*. Consistent with this pattern, one of the few, if not only, published prospective human studies assessing the microbiome and neurodevelopment reported the association of greater relative abundance of *Bacteroides* with better subsequent neurodevelopment.^11^ We did not observe any statistically significant associations of ASQ-3 scores with gut microbiome diversity, perhaps because this reductive measure does not capture other aspects of microbial communities (specific taxonomic composition and/or functional products of specific taxa) that may be most relevant to child neurodevelopment.

Exposures to gut microbes in infancy prime the development of the gut-brain axis, with potential long-term associations with neurobehavioral health.^21^ Data from experimental animal model studies show that germ-free mice (born without any gut microbiota) display altered synaptic protein expression,^22^ increased blood-brain barrier permeability,^23^ memory deficits,^24^ and social deficits with relevance to autism.^8^ A number of mechanisms have been postulated to underlie these experimental findings, including the microbiome’s central role in immune system development and regulation.^6,7^ In addition, signals to the brain that originate from the gut microbiome are likely mediated through a variety of bacterial metabolites, such as neuroactive short-chain fatty acids n-butyrate, acetate, and propionate, which bind free fatty acid receptors in the brain.^25^ Bacteria also produce neurotransmitters that may be associated with central neural pathways by interfering with host transmitter functions.^26^

Of note, poor performance of children on the ASQ-3 (particularly on communication skills) at 16 to 30 months of age has been shown to be sensitive (but not specific) for diagnosis of ASDs.^13^ Children with ASDs often have functional gastrointestinal disorders, which are thought to originate from disturbances of the microbiome gut–brain axis rather than from known physiological or anatomic abnormalities.^27^ A number of cross-sectional studies comparing the gut microbiome of neurotypical children with that in children with ASDs have reported increased levels of Clostridiales in the gut microbiome of individuals with ASDs, including higher levels of *Clostridium*,^28^ *Clostridium histolyticum*,^29^ and *Ruminococcus*.^30^ A report by Luna et al^27^ showed increased levels of Clostridiales (Lachnospiraceae and Ruminococcaceae), in children with ASDs who reported gastrointestinal pain. However, in cross-sectional studies of the gut microbiome and autism, it is difficult to determine the directionality of associations. For example, ASDs (and associated behaviors related to diet, medication, or supplement use) could alter the microbiome, or the microbiome gut–brain axis could contribute to the origin of autistic traits, their enhancement, or both. No formal ASD diagnoses were available in the VDAART cohort data, but the ability to detect associations between the infant gut microbiome and later social and communication skills in a typically developing population suggests that these prospective associations may also be present in populations at risk of more extreme phenotypes. For example, a modest decrement in mean ASQ-3 score across a predominantly neurotypical population of children can disproportionately affect the prevalence of abnormal scores and the resultant population burden of developmental disorders.^31^ In this context, even modest microbiome-associated decrements in average ASQ-3 performance have potential clinical and public health importance.

We also observed associations between the infant gut microbiome (*Bacteroides* and Enterobacteriaceae) and fine motor skills, a microbial profile that is markedly different from the associations between Clostridiales and communication and personal and social skills deficits. Less is known about potential associations between the gut microbiota and development of fine motor skills. Fine motor skills are correlated with other abilities and may serve as an indicator for cognitive skills such as visual information processing.^32^ However, higher abundancy of *Bacteroides* in infancy has been associated with better, rather than worse, cognitive development elsewhere.^11^

### Strengths and Limitations

The present study has several strengths. We identified prospective associations between the infant gut microbiome and preschool age developmental outcomes (communication, personal and social, and fine motor skills) in a multiracial/multiethnic cohort composed mainly of typical developing children. Expected associations between demographic variables (eg, maternal educational level and child sex) and development were found in our analysis, suggesting that the ASQ-3 parental evaluation was a valid tool for assessment of childhood development in our cohort.^33^ Associations between the infant gut microbiome and ASQ-3 scores were robust to adjustment for these demographic variables and other potential confounders. Although fecal flora samples were collected during the 3- to 6-month age range (mean age, 5 months), we do not believe that potential age-related changes in gut microbiota contributed to exposure misclassification in our analysis because infant age at fecal flora assessment was not a predictor of either bacterial coabundance or infant gut diversity in the VDAART study.

Some study limitations should be noted. The postnatal gut microbiome is remarkably dynamic up to 3 years of age, which is also a critical period for brain development.^22^ Assessment of the infant gut microbiome at a single time does not fully capture the early life evolution of the microbiome, which may also be important to neurocognitive outcomes. Although some of our findings (notably, that increased abundance of gut Clostridiales was associated with reduced communication and personal and social skills ASQ-3 scores) overlapped with results from studies^28,29,30^ on the gut microbiome and ASDs, we did not have data on clinical diagnoses of ASDs for our analysis and were limited by small sample size for odds ratio estimates (Table 3). The mode of ASQ-3 administration may have made assessment for some ASQ-3 domains difficult (eg, parents responded to questions about a child’s ability to perform a problem-solving task without testing the child in real time). However, daily experiences of parents with their child was likely associated with increased accuracy in other domains (such as communication skills). Although our findings were robust to adjustment for confounders, there are other factors that may be associated with gut microbiota, such as antibiotic use from infancy to 3 years of age and colonic transit time, for which we were unable to account. Leveraging data from the VDAART cohort facilitated our ability to address a question for which there have been few previous studies but also meant that our analysis was embedded in a clinical trial, a design that could influence generalizability. Adjustment for clinical site or vitamin D treatment group did not alter our findings. However, in secondary analyses, higher vitamin D supplementation during pregnancy was potentially protective of gut microbiome–associated decrements in some ASQ-3 measures. This finding suggests that our overall estimates may have underestimated adverse microbiome associations. Conversely, this finding has interesting implications regarding the potential for dietary interventions to mitigate neurodevelopmental risk.

## Conclusions

These findings suggest an association between infant gut microbiome composition and communication, personal and social,and fine motor skills at age 3 years. The majority of associations were driven by taxa within the order Clostridiales. Follow-up studies in other populations and use of more comprehensive neurocognitive assessment tools are needed to provide additional evidence for a prospective association between the infant gut microbiome and developmental outcomes in children.

## Notes

Supplement. **eMethods.** VDAART Study **eTable 1.** ASQ-3 Cut-points for Score Categories of “Typical,” “Need for Monitoring,” and “Need for Further Assessment” **eTable 2.** Bacterial Coabundance Factor Analysis (of Top 25 Taxa) for 307 VDAART Participants with Infant Stool Samples **eTable 3**. MaAsLin Analysis Results: Associations of Gut Microbiome Taxa (Those Present in at Least 10 Subjects) in Infants (Ages 3-6 Months) with Typical Communication Development on the ASQ-3 at Age 3 Years, N=307 **eTable 4**. MaAsLin Analysis Results: Association of Gut Microbiome Taxa (Those Present in at Least 10 Subjects) in Infants (Ages 3-6 Months) with Typical Personal/Social Development on the ASQ-3 at Age 3 Years, N=307 **eTable 5.** MaAsLin Analysis Results: Association of Gut Microbiome Taxa (Those Present in at Least 10 Subjects) in Infants (Ages 3-6 Months) with Typical Fine Motor Development on the ASQ-3 at Age 3 Years, N=307
