# Supplementary material for: Association of the Infant Gut Microbiome With Early Childhood Neurodevelopmental Outcomes: An Ancillary Study to the VDAART Randomized Clinical Trial
Source: JAMA Netw Open. 2019 Mar 22;2(3):e190905. doi: 10.1001/jamanetworkopen.2019.0905 (PMC6583279; doi:10.1001/jamanetworkopen.2019.0905)
Supplement: Supplement. — eMethods. VDAART Study eTable 1. ASQ-3 Cut-points for Score Categories of “Typical,” “Need for Monitoring,” and “Need for Further Assessment” eTable 2. Bacterial Coabundance Factor Analysis (of Top 25 Taxa) for 307 VDAART Participants with Infant Stool Samples eTable 3. MaAsLin Analysis Results: Associations of Gut Microbiome Taxa (Those Present in at Least 10 Subjects) in Infants (Ages 3-6 Months) with Typical Communication Development on the ASQ-3 at Age 3 Years, N=307 eTable 4. MaAsLin Analysis Results: Association of Gut Microbiome Taxa (Those Present in at Least 10 Subjects) in Infants (Ages 3-6 Months) with Typical Personal/Social Development on the ASQ-3 at Age 3 Years, N=307 eTable 5. MaAsLin Analysis Results: Association of Gut Microbiome Taxa (Those Present in at Least 10 Subjects) in Infants (Ages 3-6 Months) with Typical Fine Motor Development on the ASQ-3 at Age 3 Years, N=307 [file jamanetwopen-2-e190905-s001.pdf]

Supplementary Online Content

Sordillo JE, Korrick S, Laranjo N, et al. Association of the infant gut microbiome with early childhood neurodevelopmental outcomes: an ancillary study to the VDAART randomized clinical trial. *JAMA Netw Open*. 2019;2(3):e190905. doi:10.1001/jamanetworkopen.2019.0905

eMethods. VDAART Study

eTable 1. ASQ-3 Cut-points for Score Categories of "Typical," "Need for Monitoring," and "Need for Further Assessment"

eTable 2. Bacterial Coabundance Factor Analysis (of Top 25 Taxa) for 307 VDAART Participants with Infant Stool Samples

eTable 3. MaAsLin Analysis Results: Associations of Gut Microbiome Taxa (Those Present in at Least 10 Subjects) in Infants (Ages 3-6 Months) with Typical Communication Development on the ASQ-3 at Age 3 Years, N=307

eTable 4. MaAsLin Analysis Results: Association of Gut Microbiome Taxa (Those Present in at Least 10 Subjects) in Infants (Ages 3-6 Months) with Typical Personal/Social Development on the ASQ-3 at Age 3 Years, N=307

eTable 5. MaAsLin Analysis Results: Association of Gut Microbiome Taxa (Those Present in at Least 10 Subjects) in Infants (Ages 3-6 Months) with Typical Fine Motor Development on the ASQ-3 at Age 3 Years, N=307

This supplementary material has been provided by the authors to give readers additional information about their work.

© 2019 Sordillo JE et al. *JAMA Network Open.*

eMethods. **VDAART Study**

Pregnant women (who reported or whose partner reported allergies/asthma) were randomized (n=880) during the first trimester of pregnancy (10-18 weeks) to one of two treatment arms of a clinical trial: 4000 IU vitamin D + prenatal vitamins or 400 IU vitamin D + prenatal vitamins. Seventy infants were excluded, due to fetal or neonatal deaths (N=29), loss to follow-up during the prenatal period (N=27) or loss to follow-up after delivery (N=3). Of the 810 infants remaining, 333 infants donated stool samples.

Stool sample Collection and Sequencing of Bacterial 16S rRNA gene

Mothers were asked to collect ½ teaspoon of their infant's stool from a dirty diaper using a tongue depressor and to store the sample in their home freezer. Frozen stool samples were then brought to the clinical center by the infant's mother within 1-2 days or picked up at participants' homes within 24 hours of collection and stored at -80°C. Stool samples were not collected if the infant had taken antibiotics within the past 7 days. A total of 333 infants had stool samples (age 3-6 months) collected. DNA extractions were performed on stool samples, and the bacterial rRNA 16S gene (V3 to V5 hyper-variable regions) was amplified. Pyrosequencing data (Roche 454 Titanium) were produced by the Genome Center (TGI) at Washington University in St. Louis, MO.

Bioinformatics Pipeline for Processing of Bacterial 16S Sequences

Quality filtering, trimming, and chimera checking were performed. The quality filtering protocol used allows one mismatch in the primer and zero mismatches in the barcode. Chimera slayer software was used to remove chimeric reads. All samples that passed quality control screening were classified from phylum to genus level at a confidence threshold of 0.5 using the Ribosomal Database Project Naive Bayesian Classifier version 2.2, training set 6. The reads whose taxonomic assignments were lower than a 0.5 confidence threshold were assigned to the unclassified group. All samples were scaled to 1000 reads before analysis.

Covariates (Additional information about Race classification*)*

Infant's racial/ethnic group was derived from initial enrollment data on race/ethnicity of each biological parent. The 'African American' race/ethnicity category included infants of African American ancestry only, as well as those with mixed African and Hispanic ancestry. Infants of Native American or Asian descent were included in the 'Other' race category.

© 2019 Sordillo JE et al. *JAMA Network Open.*

Multivariate Associations

MaAsLin analytic software performs boosted, additive general linear models to detect associations between one group of data (metadata/predictors, including ASQ-3 binary outcomes for typical development vs. potential delayed development) and another group (microbial abundance of specific taxa). In this multivariate approach, all measured taxa are considered as outcomes simultaneously.

© 2019 Sordillo JE et al. *JAMA Network Open.*

eTable 1. ASQ-3 Cut-points for Score Categories of “Typical,” “Need for Monitoring,” and “Need for Further Assessment”

ASQ Domain	Typical	Need for Monitoring	Need for Further Assessment
Communication Score	> 40	30.99 - 40	<30.99
Gross Motor Score	>46	36.99 – 46	<36.99
Fine Motor Score	>32.5	18.07 – 32.5	<18.07
Problem Solving Score	>41	30.29 – 41	<30.29
Personal/Social Score	>44	35.3 - 44	<35.33

*ASQ-3=Ages and Stages Questionnaire 3rd Edition

© 2019 Sordillo JE et al. *JAMA Network Open.*

eTable 2. Bacterial Coabundance Factor Analysis (of Top 25 Taxa) for 307 VDAART Participants with Infant Stool Samples

	**Bacterial Co-abundance Factor Loadings**			
	**Factor 1**	**Factor 2**	**Factor 3**	**Factor 4**
Bacteroides	**-0.31**	-0.28	**0.70**	-0.22
Escherichia Shigella	-0.23	-0.28	**-0.44**	-0.07
Bifidobacterium	-0.28	-0.17	**-0.47**	0.00
Veillonella	-0.08	0.08	0.15	**0.66**
Blautia XIV	0.23	0.02	0.06	-0.08
Enterococcus	-0.02	0.05	-0.29	-0.11
Klebsiella	-0.05	**0.46**	-0.01	0.02
Streptococcus	-0.09	0.00	-0.20	0.18
Lachnospiraceae Dorea	**0.59**	-0.08	-0.14	-0.23
Lachnospir. Coprococcus	**0.41**	0.00	0.06	0.24
*Lachnospiraceae U.***	**0.82**	-0.02	-0.06	-0.09
Parabacteroides	-0.05	-0.17	0.28	-0.23
Collinsella	-0.03	0.05	0.08	-0.23
Lactobacillus	-0.20	-0.05	-0.12	0.15
Clostridium	0.03	0.12	-0.07	**0.36**
Coprobacillus	0.16	-0.02	-0.14	0.02
Sporacetigenium	0.14	0.06	-0.13	0.08
*Enterobacteriaceae U.***	-0.04	**0.86**	-0.06	-0.05
Enterobacter	-0.07	**0.76**	0.00	-0.17
Holdemania	0.29	-0.01	-0.03	-0.10
Megasphaera	-0.09	0.06	0.06	-0.10
Eubacterium	0.21	-0.03	0.08	0.06
Akkermansia	0.06	0.07	0.00	-0.12
*Clostridiales U.***	**0.65**	-0.02	0.07	0.01
Lactococcus	0.04	0.05	-0.03	-0.15

*VDAART=Vitamin D Antenatal Asthma Reduction Trial ****U. denotes "Unclassified" at lower taxonomic levels*

© 2019 Sordillo JE et al. *JAMA Network Open.*

eTable 3. MaAsLin Analysis Results: Associations of Gut Microbiome Taxa (Those Present in at Least 10 Subjects) in Infants (Ages 3-6 Months) with Typical Communication Development on the ASQ-3 at Age 3 Years, N=307

Feature			Coefficient	N not 0**	p value	q Value (FDR adjusted p value)
Order	Family	Genus				
Flavobacteriales	Weeksellaceae	Chryseobacterium	0.002759	20	0.013	0.566475
Clostridiales	Veillonellaceae	Acidaminococcus	-0.01418	14	0.015634	0.566475
Enterobacteriales	Enterobacteriaceae	Salmonella	-0.00271	22	0.015689	0.566475
Lactobacillales	Enterococcaceae	Unclassified	-0.0118	135	0.019703	0.566475
Enterobacteriales	Enterobacteriaceae	Erwinia	-0.00659	123	0.036579	0.584345
Lactobacillales	Unclassified		-0.00695	126	0.032388	0.584345
Clostridiales	Lachnospiraceae	Ruminococcus	-0.06541	271	0.04065	0.584345
Clostridiales	Christensenellaceae	Christensenella	-0.0026	10	0.036947	0.584345

*Nominally significant features (P < 0.05) are reported. Analysis adjusted for gestational age, race, gender, breast feeding in 1st 6 months of life, c-section, age at ASQ, maternal education, maternal marital status, maternal age, low income (<$30,000), antibiotics in the first days of life, treatment group and clinical site **Number of participants with detectable feature

© 2019 Sordillo JE et al. *JAMA Network Open.*

eTable 4. MaAsLin Analysis Results: Association of Gut Microbiome Taxa (Those Present in at Least 10 Subjects) in Infants (Ages 3-6 Months) with Typical Personal/Social Development on the ASQ-3 at Age 3 Years, N=307

**Feature**			**Coefficient**	**N not 0****	**p value**	**q Value (FDR adjusted p value)**
**Order**	**Family**	**Genus**				
Clostridiales	Lachnospiraceae	Oribacterium	-0.00235	16	0.003331	0.383099
Clostridiales	Ruminococcaceae	Oscillospira	-0.03631	218	0.007523	0.432552
Lactobacillales	Enterococcaceae	Unclassified	-0.01199	135	0.021813	0.627134
Lactobacillales	Leuconostocacea	Weissella	-0.0031	23	0.020977	0.627134
Clostridiales	Lachnospiraceae	Ruminococcus_	-0.07184	271	0.029383	0.675807
Enterobacteriales	Enterobacteriaceae	Salmonella	-0.00235	22	0.042865	0.821583

***** Nominally significant features (P < 0.05) are reported. Analysis adjusted for gestational age, race, gender, breast feeding in 1st 6 months of life, c-section, age at ASQ, maternal education, maternal marital status, maternal age, low income (<$30,000), antibiotics in the first days of life, treatment group and clinical site **Number of participants with detectable feature

© 2019 Sordillo JE et al. *JAMA Network Open.*

eTable 5. MaAsLin Analysis Results: Association of Gut Microbiome Taxa (Those Present in at Least 10 Subjects) in Infant (Ages 3-6 Months) with Typical Fine Motor Development on the ASQ-3 at Age 3 Years, N=307

**Feature**			**Coefficient**	**N not 0****	**p value**	**q Value (FDR adjusted p value)**
**Order**	**Family**	**Genus**				
Enterobacteriales	Enterobacteriaceae	Klebsiella	-0.03339	239	0.000312	0.035843
Enterobacteriales	Enterobacteriaceae	Salmonella	-0.00268	22	0.001024	0.058892
Clostridiales	Veillonellaceae	Megasphaera	-0.00418	147	0.018578	0.517589
Coriobacteriales	Coriobacteriaceae	Atopobium	0.003611	100	0.020417	0.517589
Actinomycetales	Actinomycetaceae	Actinomyces	0.006387	220	0.033323	0.517589
Clostridales	Veillonellaceae	Phascolarctobacterium	-0.01288	32	0.035419	0.517589
Enterobacteriales	Enterobacteriaceae	Erwinia	-0.00472	123	0.040415	0.517589
Lactobacillales	Lactobacillaceae	Lactobacillus	0.002866	147	0.040478	0.517589
Lactobacillales	Streptococcaceae	Streptococcus	0.031244	307	0.040507	0.517589

*Nominally significant features (P < 0.05) are reported. Analysis adjusted for gestational age, race, gender, breast feeding in 1st 6 months of life, c-section, age at ASQ, maternal education, maternal marital status, maternal age, low income (<$30,000), antibiotics in the first days of life, treatment group and clinical site **Number of participants with detectable feature

© 2019 Sordillo JE et al. *JAMA Network Open.*
